# The impact of physical activity on academic burnout in Chinese college students: a moderated chain mediation model

**DOI:** 10.3389/fpsyg.2025.1681455

**Published:** 2025-10-22

**Authors:** Jiang Xiang, Yun Jiang

**Affiliations:** ^1^Sports College, Zhangjiajie College, Zhangjiajie, Hunan, China; ^2^Mental Health Education Center, Zhangjiajie College, Zhangjiajie, Hunan, China

**Keywords:** physical activity, academic burnout, social support, psychological capital, gender differences

## Abstract

**Background:**

Academic burnout has become increasingly prevalent among college students under multiple stressors. While earlier studies indicate that physical activity may be associated with lower levels of academic burnout, the specific processes involved remain insufficiently clarified.

**Purpose:**

Guided by stress-coping theory and gender role theory, this study examined the potential chain mediation effects of social support and psychological capital on the relationship between physical activity and academic burnout among college students, as well as the moderating role of gender.

**Methods:**

Using a random sampling method, a total of 858 valid questionnaires were collected, with male students accounting for 48.6% of the sample. Data were collected using standardized scales on college students’ perceptions of the study variables, with age and grade controlled for in the analyses, and the relationships among these variables were analyzed using bootstrapping methods.

**Results:**

The results indicated a significant negative correlation between physical activity and academic burnout. Both social support and psychological capital individually mediated this relationship, accounting for 14.5 and 10.1% of the total effect, respectively, while their combined chain mediation effect accounted for 23.2%. Furthermore, gender differences were observed: female students demonstrated a stronger effect of physical activity on enhancing social support, whereas male students exhibited a more pronounced effect of physical activity in directly mitigating academic burnout.

**Conclusion:**

Overall, this study highlights plausible pathways through which physical activity may relate to academic burnout by fostering social support and psychological capital. These findings expand the current understanding of the association between physical activity and academic burnout, and offer valuable implications for educational practice.

## 1 Introduction

Derived from the concept of “occupational burnout,” academic burnout (AB) refers to the state of feeling exhausted due to academic pressures, adopting a cynical and detached attitude toward their studies, and experiencing a diminished sense of competence as a student (Maslach and Jackson, 1981; Zhang et al., 2007). Currently, AB is both widespread and severe among Chinese college students. A large-scale survey involving nearly 23,000 college students revealed that approximately 60% of Chinese university students are experiencing varying degrees of AB (Liu X. H. et al., 2023). This phenomenon is associated with adverse outcomes students’ academic development, including decreased learning motivation, lower academic performance, heightened the risk of dropout, and hindered personal growth (Abreu Alves et al., 2022; Madigan and Curran, 2021; Serafica and Muria, 2023).

To understand and potentially alleviate AB, scholars have examined a range of internal and external correlates. Internal factors mainly encompass aspects such as self-efficacy, psychological resilience, physical activity, psychological capital, mental health, and self-management abilities (Kordzanganeh et al., 2021; Liu et al., 2024; ÖZhan, 2021; Rehman et al., 2024; Tran et al., 2023; Wang et al., 2021), while external factors encompass social support, family cohesion, external expectations, and campus climate (Bai et al., 2025; Kim and Lee, 2022; Yu et al., 2021). Building on this extensive body of research, scholars have suggested that enhancing learning strategies—such as adopting Teacher–Student Collaborative Assessment or implementing mindfulness-based interventions—may foster academic engagement and reduce the risk of AB (Jiang et al., 2025; Reyes-de-Cózar et al., 2023; Wang et al., 2024).

Physical activity (PA), recognized for its affordability and effectiveness in promoting health, has been consistently linked with psychological benefits such as enhanced self-confidence and greater resilience, which in turn are associated with improved learning performance and lower level of AB (Chen et al., 2022). Social support (SS), has been found to provide emotional, informational, and material assistance to students, which may buffer stress and correlate with reduced AB (Fu, 2024; Kim and Lee, 2022). Psychological capital (PsyCap) has also been identified as a predictor of academic engagement, which is in turn related to lower burnout and fatigue (Virga et al., 2020). Nevertheless, these associations appear to vary across sociocultural contexts, physiological characteristics, and behavioral patterns. For example, male and female students often differ in their motivation, type, and frequency of engaging in PA (Espada et al., 2023), suggesting that gender may shape the extent to which PA relates to SS, PsyCap, and AB.

Although the effects of PA, SS, PsyCap, and gender on AB among college students have been extensively explored, no study has yet integrated these factors to specifically examine their combined influence on AB. Therefore, this study proposes the following research questions:

1. How do SS and PsyCap mediate the relationship between PA and AB among college students?
2. Does gender moderate the mediating effect of social support and psychological capital on the relationship between physical activity and academic burnout?

To answer these questions, the study proposes a chain mediation model incorporating PA, SS, PsyCap, and AB, while also analyzing the moderating role of gender. The model specifies plausible pathways grounded in stress-coping and gender role theories, but the cross-sectional design precludes causal inference. Thus, the proposed associations should be regarded as tentative, requiring further longitudinal or experimental research to verify their robustness across different cultural and institutional contexts. Despite these limitations, this study allows for a more detailed exploration of the dynamic associations among PA, SS, PsyCap, and AB, thereby providing strong theoretical support for educational institutions and educators in their efforts to mitigate AB among college students.

### 1.1 Theoretical foundation

According to the stress-coping theory proposed by Lazarus and Folkman (1984), psychological stress results from ongoing exchanges between individuals and their environments, which simultaneously shape both the perception of stress and the coping mechanisms adopted. Stress arises when an individual perceives a demand as significant but beyond their available coping resources (Folkman, 2013). In stressful situations, individuals appraise the circumstances and mobilize coping resources based on this appraisal (Lazarus, 1993). When students experience academic stress that exceeds their coping capacity, they are likely to report emotional exhaustion, diminished self-efficacy, and other negative emotional states (Ito and Brotheridge, 2003). Without adequate coping resources, stress may remain unresolved, leaving college students in a prolonged state of distress and leading to AB (Maslach et al., 1997). In this study, PA is conceptualized as a coping strategy that may help students manage academic stress and, consequently, be linked with lower levels of AB (Park and Iacocca, 2014). By providing essential coping capacities, SS and PsyCap have been associated with alleviating the detrimental consequences of stress, aiding students in managing their emotions and improving their ability to meet academic requirements (Avey et al., 2009; Thoits, 1995).

As proposed by gender role theory, societal norms tied to gender are thought to influence behavioral patterns and emotional reactions in individuals (Eagly et al., 2000). The process of socialization leads to gender-specific coping tendencies, with problem-solving more commonly observed among males, and emotional regulation more frequently reported among females when confronting stressful situations (Hobfoll et al., 1994). Based on this theory, males and females may display somewhat different coping patterns when facing academic stress. For example, in some contexts, females may rely more heavily on emotional support and social interaction to alleviate stress, whereas males may prefer PA or independent problem-solving to manage challenges. Therefore, gender is likely to moderate the extent to which PA, SS, and PsyCap are associated with AB.

### 1.2 Physical activity and academic burnout

Physical activity generally refers to engaging in regular bodily movements aimed at promoting overall health and emotional stability (Carrapatoso et al., 2022; Wang, 2021). A notable expansion of empirical studies has underscored the contribution of PA to the psychological well-being of college student populations. Empirical findings suggest that engaging in PA is associated with reduced psychological difficulties, including anxiety and depression (Magro et al., 2023), and may be linked to lower levels of AB. Several studies have reported associations between PA and decreased AB (Chen et al., 2022; Jin et al., 2024; Rehman et al., 2024; Rosales-Ricardo and Ferreira, 2022). Chen et al. (2022), in their investigation of determinants influencing students’ academic stress, identified PA as an important correlate of stress, with regularly active students reporting lower academic strain. Regular PA has also been associated with better emotional well-being and lower AB, as students who consistently engage in PA benefit from both physical and psychological recovery that may buffer feelings of exhaustion and pressure related to academic work (Chen and Zhang, 2022; Prontenko et al., 2020; Zhang, 2023). According to Fu et al. (2023), frequent engagement in PA was identified as a significant protective factor against AB among adolescents, facilitating a more constructive outlook when encountering academic pressures. Moreover, Naczenski et al. (2017), through a meta-analysis of 10 longitudinal or intervention studies, found that PA exerted a significant impact on reducing burnout, particularly by strongly alleviating exhaustion. In addition, the protective role of PA against AB has been confirmed across different cultural contexts, such as Iran (Khosravi, 2021), the United Kingdom (Lemola et al., 2021), and the United States (Boone et al., 2023). Longitudinal studies have further supports these associations. Ye et al. (2024) conducted a two-month longitudinal survey and found that PA alleviated AB, which in turn was associated with reduced depressive symptoms, with the full lagged mediating effect of AB being stronger among students engaged in high-intensity PA. Therefore, the following hypothesis is proposed:

*H1*: PA is hypothesized to show a significant negative association with AB among college students.

### 1.3 The mediating role of social support

Social support encompasses both emotional reassurance and tangible assistance provided by significant others, including family and friends (Zimet et al., 1988). Within the social lives of college students, support from personal social networks (e.g., family, community) primarily offers emotional support, while support from institutional networks (e.g., peers, teachers) mainly provides informational support (Mishra, 2020). Social support is obtained through individuals’ interactions and communications with their external environment, and PA offers students valuable opportunities to build connections with others (Restubog et al., 2010). In particular, participation in group or team sports has been associated with strengthened emotional bonds and cooperation through shared participation, thereby fostering friendships and support networks that enhance their emotional support and social resources (Li and Zhang, 2025; Maher et al., 2024; Shu et al., 2024; Yan et al., 2025). Previous studies have suggested that SS may function as a mediator between PA and AB. As a critical coping resource, SS has been linked with lower levels of AB by providing various forms of assistance to college students (Kim and Lee, 2022; Kuokkanen et al., 2024; Liu Z. et al., 2023; Ye et al., 2021). Based on a questionnaire study, Liu Z. et al. (2023) found that SS from teachers and peers was positively related to academic engagement, which in turn was associated with lower AB. Additionally, Gao et al. (2025) observed that as adolescents reporting higher levels of PA also perceived greater external support, which was associated with lower AB. Although these associations are consistent, they may vary depending on contextual factors such as cultural norms, the type of PA, or whether SS is assessed as perceived or received. However, to date no systematic review or meta-analysis has comprehensively synthesized this evidence, and the mediating role of SS should therefore be regarded as provisional. Therefore, the following hypothesis is proposed:

*H2*: SS is hypothesized to mediate the association between PA and AB.

### 1.4 The mediating role of psychological capital

Psychological capital entails a positively oriented psychological condition that incorporates four key elements: self-belief, optimism, hopeful thinking, and resilience (Luthans, 2012; Newman et al., 2014). The theory of PsyCap emphasizes that these psychological resources are not static but can be enhanced through external interventions and personal efforts (Luthans and Youssef-Morgan, 2017). In academic contexts, PsyCap can be cultivated through structured feedback, incremental challenges that promote mastery, and mentoring relationships that provide guidance and role modeling (Luthans, Avolio, et al., 2007a). These processes directly reinforce self-efficacy, optimism, resilience, and hope. PA complements these mechanisms by offering mastery experiences, social bonding opportunities, and a context for incremental goal-setting (Bailey et al., 2013), thereby strengthening PsyCap in ways that extend beyond the classroom. Students who regularly engage in PA may experience physical recovery that is associated with better psychological well-being. Exercise has been linked to neurochemical processes such as endorphin release, which are related to improved mood, reduced anxiety and depression, and greater confidence and optimism (Mikkelsen et al., 2017). Moreover, consistent PA has been associated with increased confidence and self-esteem, enhanced self-control, and strengthened stress resilience, which together may contribute to the development of PsyCap (Li et al., 2024; Wei et al., 2024; Zhao H. et al., 2025). The elements of PsyCap help individuals maintain a constructive mental state under adverse circumstances and are linked with stronger problem-solving capacity (Avey et al., 2009; Hazan Liran and Miller, 2019). Consequently, students with higher levels of PsyCap are therefore more likely to report stronger emotional regulation and self-efficacy, which allows them to better alleviate negative emotions when facing academic stress, thus experiencing lower levels of AB (Nabais et al., 2024; Tang, 2024). Although these findings are promising, meta-analytic coverage of PsyCap’s mediating role remains absent. Future research should evaluate this mechanism more systematically and also consider how cultural norms and academic environments may shape these associations. Therefore, the following hypothesis is proposed:

*H3*: PsyCap is hypothesized to mediate the association between PA and AB.

### 1.5 The chain mediating role of social support and psychological capital

Regular participation in PA may provide college students with increased opportunities for social engagement. Supportive interactions with instructors and peers have been associated with stronger perception of SS (Shu et al., 2024). As a valuable external resource, SS has been linked to enhanced PsyCap traits—such as confidence, optimism, resilience, and hope—through both emotional comfort and tangible assistance (Deng and Wang, 2024; Zhou et al., 2024). Support from family members, friends, or classmates may foster a sense of security and stability within one’s social network, which in turn may be related to stronger psychological resilience and self-efficacy (Labrague, 2024; Zhang X. et al., 2021). Moreover, the emotional comfort provided by SS is often associated with greater optimism and hope when facing challenges, thereby promoting overall PsyCap. Previous researches have demonstrated an association between SS and PsyCap (Siu et al., 2023; Wang et al., 2023). Individuals with higher levels of PsyCap are more likely to adopt adaptive coping strategies under academic pressure, which has been associated with lower levels of AB (Nabais et al., 2024). According to Lin et al. (2025), regular engagement in PA by Chinese college students was positively related to perceived SS, which was subsequently linked to higher PsyCap and improved academic advancement. In addition, Deng and Wang (2024), through a survey of 874 Chinese college students, found that PA was positively associated with perceived SS, which in turn was linked to elevated PsyCap and lower social anxiety. Although current research has examined in detail the impact of PA on AB, the underlying mechanisms remain insufficiently clarified. To date, no study has explicitly demonstrated whether PA enhances college students’ perceptions of SS, thereby strengthening their social capital and ultimately mitigating AB. To address this gap in traditional research models, the present study constructs a comprehensive chain mediation framework with the aim of extending the current understanding of the interconnections among these variables. Therefore, the following hypothesis is proposed:

*H4*: SS and PsyCap are hypothesized to jointly mediate the association between PA and AB.

### 1.6 The moderating role of gender

First, the effect of PA on SS may vary by gender. As outlined by gender role theory (Eagly and Wood, 1999), social norms are thought to encourage men to display competitiveness and aggressiveness, while women are expected to exhibit affiliation and emotional regulation. These expectations may lead men to participate more frequently in physically demanding competitive sports, such as basketball and soccer, which have been associated with instrumental social support by enhancing peer interactions and strengthening team cohesion (Peng et al., 2025). In contrast, women are often more prefer non-competitive activities such as yoga and dance, which emphasize emotional exchange and intimacy, thereby offering greater emotional support (Deng et al., 2023; Shu et al., 2024). Therefore, even at similar levels of PA, men and women may acquire different forms of SS, suggesting gender’s role in moderating the association between PA and SS.

Second, the impact of PA on PsyCap may also be moderated by gender. Psychological capital consists of self-efficacy, optimism, resilience, and hope, and individuals’ sensitivity to these psychological resources during PA may differ by gender. Engagement in PA may provide males with opportunities to encounter challenges, such as in competitive, performance-oriented PA types like team sports or strength training, and gain mastery experiences, which are often linked to higher confidence and psychological resilience (Lirgg, 1991). This pattern partly stems from gendered social expectations that encourage men to pursue activities emphasizing achievement and overcoming obstacles (van Uffelen et al., 2017). Females, on the other hand, may place greater emphasis on emotional regulation and self-care derived from PA, often through low-intensity, socially oriented PA types like group yoga or community fitness classes, where social exposure to peers and emotional interaction during group sessions strengthen social bonding. Such social and emotional benefits from PA exert a stronger influence on optimism and hope in females (Zhao G. et al., 2025), again reflecting how social expectations shape the link between PA and specific PsyCap components. Thus, the pathway through which PA enhances PsyCap and subsequently reduces AB may also differ in strength depending on gender: for males, mastery-driven and competitive PA tends to yield greater benefits, as this type of activity boosts their self-efficacy and resilience; for females, socially focused and emotionally oriented PA is more advantageous, as it strengthens their optimism and hope.

Furthermore, the direct association between PA and AB may also vary by gender. For many men, PA often serves as a primary strategy for stress relief and emotional regulation, which has been linked to lower AB (van Uffelen et al., 2017). Unlike men, women’s coping with academic stress tends to involve greater reliance on SS networks and emotional exchanges, which may reduce the direct benefits of PA on AB unless accompanied by additional support interventions (Eschenbeck et al., 2007). Additionally, differing societal expectations regarding the academic roles of male and female students may further contribute to gender-based variations in the PA–AB association. The moderating role of gender has also been confirmed across different national and cultural contexts. For example, González et al. (2023), in a study of Latino college students in the United States during the COVID-19 pandemic, found that the negative association between PA and AB was significant only among female students, whereas no such relationship was observed in the overall sample or in the male subgroup.

In summary, gender serves as an important moderating variable that may influence the mechanisms by which PA, through SS and PsyCap, is associated with AB. Based on this reasoning, the study puts forward these hypotheses:

*H5a*: Gender is hypothesized to moderate the association between PA and SS.

*H5b*: Gender is hypothesized to moderate the association between PA and PsyCap.

*H5c*: Gender is hypothesized to moderate the association between PA and AB.

### 1.7 The current study

**Figure 1:**
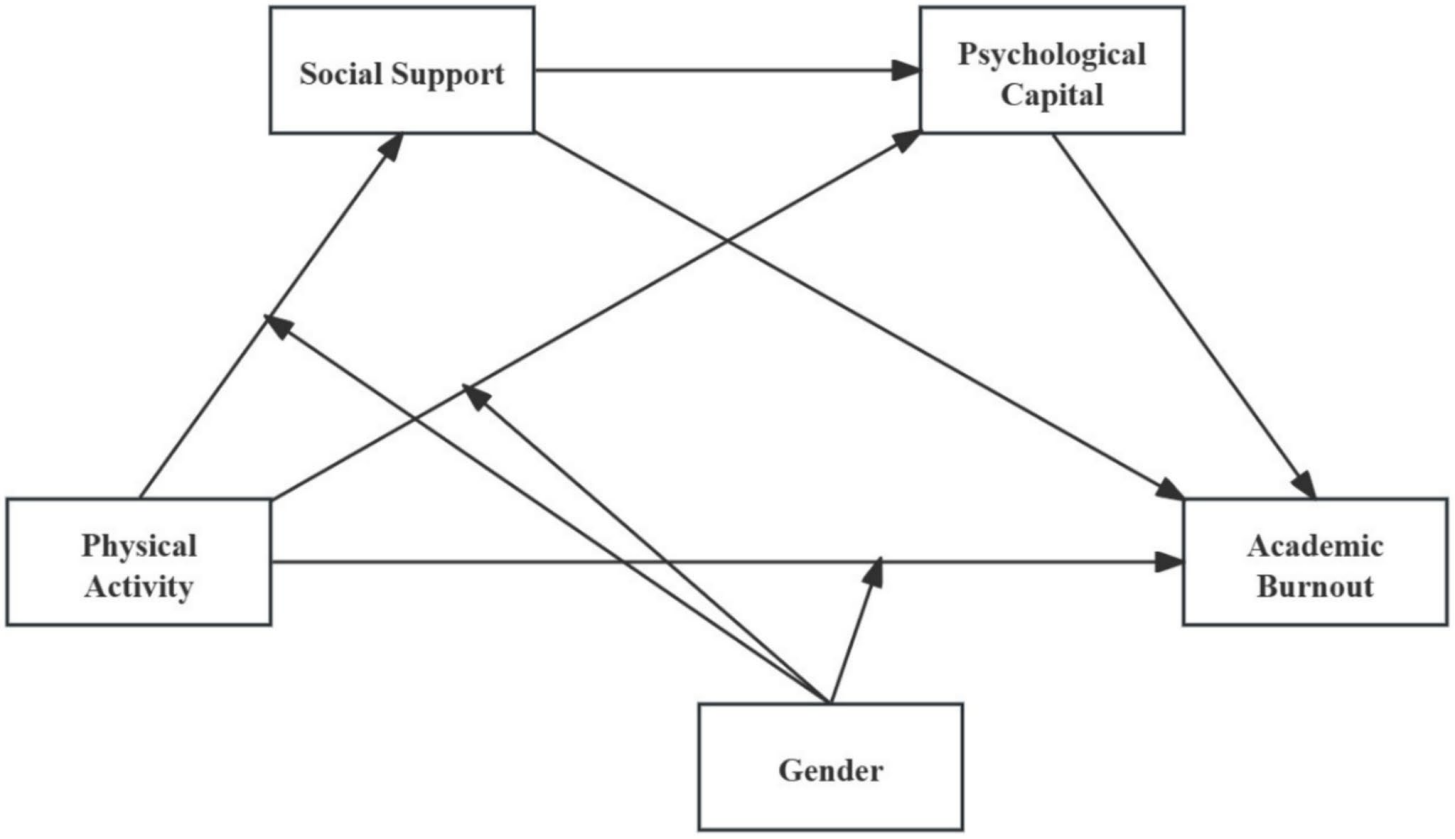
Research model.

Although numerous studies have examined the relationship between PA and AB, integrated models incorporating the serial mediation of SS and PsyCap and assessing gender’s moderating effect remain underexplored. Therefore, based on the stress-coping theory and gender role theory, and in accordance with the above hypotheses, the research team has developed the research model presented in Figure 1.

## 2 Methods

### 2.1 Samples and data collection

Utilizing random sampling, the study surveyed students from three universities in Hunan Province, China. Data collection spanned from September to October 2024. The research team first contacted university administrators to obtain basic information on departments, year levels, classes, and class sizes. Given that the student populations of all three universities exceed ten thousand, to ensure a relatively balanced number of participants across the universities, we randomly selected 10 classes from each university as the study sample. To guarantee that students from different grades and departments were represented, the research team renumbered all classes from the three universities and then used the random number generator in SPSS to randomly select 30 classes. Researchers then contacted class instructors and distributed the survey during regular class sessions. The questionnaire was disseminated through the Wenjuanxing platform,^1^ with recruitment conducted via social media, email, and institutional networks. Anonymity and confidentiality were maintained throughout the process. Students who were taking medication for psychological health issues or who suffered from medical or chronic physical conditions that limited their ability to engage in PA—particularly vigorous activity—were excluded. Including such participants could have biased the results by underestimating the role of PA in alleviating AB. Prior to participation, all respondents gave informed consent. This study was reviewed and approved by the Zhangjiajie College (Approved No. ZC-IRB-2024-009). All procedures involved in this study were carried out strictly in accordance with relevant ethical guidelines and regulations, including the Declaration of Helsinki.

The research team distributed a total of 973 questionnaires and collected 887 responses. After screening and verification, 29 invalid questionnaires were deleted. Specifically, 6 questionnaires were excluded due to incompleteness (answering rate below 80% with too many missing items to allow subsequent statistical analysis), 9 questionnaires were excluded because the response time was either too short or too long (e.g., less than 1 min or more than 30 min), and 14 questionnaires were excluded because the same option was selected for too many items (e.g., more than 80% of items marked as “strongly disagree” or “strongly agree”). In the end, 858 valid questionnaires were retained, yielding a valid response rate of 88.2%. To examine whether these exclusions caused systematic bias, we compared the included and excluded samples on basic demographic characteristics (gender, age, grade). The results showed no significant differences between the two groups (all *p* > 0.05), indicating that the exclusions did not materially bias the composition of the final sample.

An overview of the demographic details of the participants is shown in Table 1. This study only collected three demographic variables: gender, age, and grade. Other information (e.g., socioeconomic status, major, GPA, weekly study hours) was not collected, primarily to reduce socially desirable responding, protect participants’ privacy, and decrease questionnaire length and response burden (Larson, 2019). These three demographic variables were included as covariates in all regression and mediation models. No missing values were present for these variables. The gender distribution was relatively balanced, with 417 male participants (48.6%) and 441 female participants (51.4%). The sample was predominantly composed of individuals aged 18 to 22 (92.8%), with the majority (60.9%) clustered within the 18–20 years age category. In terms of academic year, participants were fairly evenly distributed across year levels, though senior students represented a smaller proportion, accounting for only 15.1% of the sample.

**Table 1:** Demographic characteristics of the sample.

Categories	Subgroups	Included (*n* = 858)	Excluded (*n* = 29)	*p*
Gender	Male	417 (48.6%)	13 (44.8%)	0.833
	Female	441 (51.4%)	16 (55.2%)	
Age	Under 18	44 (5.1%)	3 (10.3%)	0.442
	18–20	522 (60.9%)	19 (65.5%)	
	20–22	274 (31.9%)	7 (24.2%)	
	Over 22	18 (2.1%)	0 (0%)	
Grade	Freshmen	239 (27.9%)	6 (20.7%)	0.648
	Sophomores	237 (27.6%)	11 (37.9%)	
	Juniors	252 (29.4%)	8 (27.6%)	
	Seniors	130 (15.1%)	4 (13.8%)	

### 2.2 Measures

Physical activity was measured using the Physical Activity Rating Scale-3 (PARS-3), adapted by Liang (1994). The scale includes three self-reported items evaluating the intensity (“*How intense is your physical exercise?*”), duration (“*How many minutes do you spend on each of the above exercises?*”), and frequency (“*How many times do you engage in these activities each month?*”) of PA, with each scored on a five-point scale (1–5). The total PA score was calculated using the following formula: Total score = Intensity × (Duration − 1) × Frequency. The total score ranges from 0 to 100, and PA levels were categorized as low (≤19), moderate (20–42), and high (≥43). The PARS-3 scale demonstrated good psychometric properties, with a Cronbach’s alpha of 0.706, a KMO of 0.621, and a significant Bartlett’s test of sphericity (*p* < 0.001).

Academic burnout was assessed using the Learning Burnout Scale developed by Lian et al. (2005), which has been widely validated in Chinese university populations (Zhang J. Y. et al., 2021). The scale includes 20 items covering three dimensions: emotional exhaustion (8 items, e.g., *“I feel emotionally drained from my studies”*), improper behavior (6 items, e.g., *“I have become less interested in my studies since I enrolled in this program”*), and reduced sense of accomplishment (6 items, e.g., *“I can effectively solve the problems that arise in my studies”* – reverse-scored). Eight items are reverse-scored, while twelve are positively scored. Using a 5-point Likert format, the scale generates cumulative scores from 20 to 100, where elevated scores signify higher degrees of AB. With a Cronbach’s alpha of 0.924, KMO value of 0.949, and significant Bartlett’s sphericity test (*p* < 0.001), the scale exhibited excellent reliability and sound construct validity. The results of confirmatory factor analysis (CFA) were: *χ^2^/df* = 3.864, RMSEA = 0.058, GFI = 0.934, AGFI = 0.911, IFI = 0.940, CFI = 0.940, and TLI = 0.926.

Social support was measured using the Multidimensional Scale of Perceived Social Support (MSPSS) developed by Zimet et al. (1988). The Chinese version of this scale has been validated in previous studies (Chou, 2000) and has demonstrated good reliability and validity among college student populations (Huang and He, 2025). This scale contains 12 items across three dimensions: support from friends (e.g., *“My friends really try to help me”*), family (e.g., *“My family is willing to help me when I need it”*), and significant others (e.g., *“There is a special person who is around when I am in need”*), with four items per dimension. Participants self-rated their perceived support using a 5-point Likert scale, producing a total score ranging from 12 to 60, with higher scores reflecting greater perceived SS. The MSPSS exhibited solid psychometric qualities, demonstrated by Cronbach’s alpha of 0.941, KMO of 0.949, and a significant Bartlett’s sphericity test (*p* < 0.001). CFA results indicated good model fit: *χ^2^/df* = 3.835, RMSEA = 0.058, GFI = 0.970, AGFI = 0.945, IFI = 0.984, CFI = 0.984, and TLI = 0.974.

Psychological capital was assessed using the PsyCap Questionnaire (PCQ) revised by Luthans et al. (2007b). The Chinese version of this scale has been validated for use among college students (Kang et al., 2021). This scale measures four components: self-efficacy (e.g., *“I feel confident in analyzing long-term problems and finding solutions”*), hope (e.g., *“When I encounter difficulties in life or academics, I can think of many ways to overcome them”*), optimism (e.g., *“I have difficulty recovering and moving on when I encounter setbacks in life”*—reverse-scored), and resilience (e.g., *“In life, I always believe that behind the darkness there is light, so there is no need to be pessimistic”*). The scale consists of 16 items, with 14 positively scored and 2 reverse-scored items. A 5-point Likert scale is applied to all items, producing total scores ranging from 16 to 80, with greater scores representing higher PsyCap. With a Cronbach’s alpha of 0.953, a KMO value of 0.970, and significant findings from Bartlett’s sphericity test (p < 0.001), the scale demonstrated strong reliability and structural validity for use in this study. CFA results indicated good model fit: *χ^2^/df* = 4.099, RMSEA = 0.060, GFI = 0.943, AGFI = 0.919, IFI = 0.970, CFI = 0.970, and TLI = 0.963.

In this study, total scores rather than subscale scores were used in the statistical analyses. This decision was made because the primary aim was to examine the relationships among PA, SS, PsyCap, and AB at an overall level rather than focusing on dimensional differences. In addition, reliability analyses indicated high internal consistency for each scale, supporting the use of total scores as overall indicators.

### 2.3 Statistical analyses

The dataset was analyzed using SPSS 26.0. Descriptive statistics were first generated, followed by Spearman correlation analyses to identify relationships among PA, SS, PsyCap, and AB. To evaluate possible common method bias, Harman’s single-factor analysis was conducted. Model fit for the theoretical framework was assessed using AMOS. Mediation analyses utilized Model 6 of the SPSS PROCESS Macro (version 4.2), and the moderating impact of gender within the chain mediation model was evaluated with Model 85.

## 3 Results

### 3.1 Descriptive statistics and correlation analysis

Table 2 presents the kurtosis (K), skewness (S), means (M), standard deviations (SD), and Spearman correlation results for all study variables. The mean score for PA was 22.140 (SD = 22.428), indicating that participants, on average, reported a relatively low-to-moderate level of PA. This result may be related to the balanced distribution of participants across different academic years, with a relatively higher proportion of junior and senior students. The higher education curriculum in China mandates physical education for freshmen and sophomores, but this requirement is generally absent for juniors and seniors. Furthermore, junior and senior students often face more demanding academic workloads and participate in internships and other forms of social practice, which may limit both the time and frequency available for PA. The greater proportion of participants from these academic cohorts may have influenced the overall decline in physical activity scores within the sample.

**Table 2:** Variable correlation test.

Variables	K	S	M	SD	1	2	3	4
1. Physical activity	0.627	1.171	22.140	22.428	1			
2. Academic burnout	1.238	0.037	53.592	11.293	−0.418***	1		
3. Social support	1.400	−0.603	42.276	8.317	0.276***	−0.564***	1	
4. Psychological capital	2.566	−0.685	55.591	10.393	0.341***	−0.624***	0.786***	1

*N* = 858; ***p < 0.001; K, Kurtosis; S, Skewness; M, Mean; SD, Standard deviation.

The Pearson correlation analysis revealed that PA (*r* = −0.418, *p* < 0.001), SS (*r* = −0.564, *p* < 0.001), and PsyCap (*r* = −0.624, *p* < 0.001) were all significantly negatively correlated with AB. PA was significantly positively correlated with PsyCap (*r* = 0.341, *p* < 0.001) and SS (*r* = 0.276, *p* < 0.001). Additionally, SS and PsyCap were strongly positively correlated (*r* = 0.786, *p* < 0.001).

### 3.2 Demographic difference analysis

To examine whether differences existed between male and female students across the study variables, independent-sample t-tests were conducted, and the results are presented in Table 3. As shown in Table 3, significant gender differences were found in PA, AB, and SS. Male students reported significantly higher levels of PA, whereas female students perceived stronger AB and SS. No significant gender difference was observed for PsyCap.

**Table 3:** Differences of gender variables in the physical exercise, academic burnout, social support and psychological capital.

Variables	Total (*n* = 858; M ± SD)	Male (*n* = 417; M ± SD)	Female (*n* = 441; M ± SD)	*t*	*p*
PA	22.140 ± 22.428	29.45 ± 24.146	15.24 ± 18.171	9.772	<0.001
AB	53.592 ± 11.293	52.813 ± 11.780	54.329 ± 10.774	−1.964	0.049
SS	42.276 ± 8.317	41.329 ± 8.612	43.172 ± 7.934	−3.256	0.001
PsyCap	55.591 ± 10.393	55.667 ± 11.096	55.519 ± 9.695	0.208	0.836

M, Mean; SD, Standard deviation; PA, Physical Activity; AB, Academic Burnout; SS, Social Support; PsyCap, Psychological Capital.

### 3.3 Common method bias

To address potential common method bias due to self-reported data, Harman’s single-factor test was performed. Seven factors with eigenvalues exceeding one emerged, with the first factor explaining 39.429% of variance, which is below the 50% threshold (Podsakoff and Organ, 1986), indicating no serious concern for common method bias.

### 3.4 Confirmatory factor analysis

To evaluate the model fit in this study, CFA was conducted. The results are presented in Table 4. The fit indices of the research model met the recommended thresholds suggested in previous studies (Marsh and Hocevar, 1985).

**Table 4:** Model fit indices.

Fit index	Final model	Reference value
*χ*^2^/*df*	2.499	<5
CFI	0.936	>0.8
TLI	0.930	>0.8
RMSEA	0.042	<0.08
SRMR	0.048	<0.08
NFI	0.898	>0.8
GFI	0.873	>0.8

χ^2^/df, Chi-square divided by Degrees of Freedom; CFI, Comparative Fit Index; TLI, Tucker-Lewis Index; RMSEA, Root Mean Square Error of Approximation; SRMR, Standardized Root Mean Square Residual; GFI, Goodness of Fit Index; IFI, Incremental Fit Index; AGFI, Adjusted Goodness of Fit Index.

### 3.5 Mediation effect analysis

This study employed Model 6 of the SPSS PROCESS macro to conduct a sequential mediation analysis using data from 858 participants. After adjusted for gender, age, grade, the results of the regression analysis were presented in Tables 5, 6.

**Table 5:** Regression analysis between the variables.

Outcome variable	Predictor variable	*β*	SE	T	Bootstrap 95% CI		R^2^	F
					LLCI	ULCI		
SS	PA	0.129***	0.013	9.883	0.103	0.154	0.120	29.146
PsyCap	PA	0.053***	0.011	4.759	0.031	0.074	0.638	299.983
	SS	0.952***	0.028	34.635	0.898	1.005		
AB	PA	−0.120***	0.015	−8.006	−0.149	−0.090	0.452	117.060
	SS	−0.260***	0.057	−4.55 9	−0.372	−0.148		
	PsyCap	−0.431***	0.046	−9.399	−0.520	−0.341		

***p < 0.001; β, Beta Coefficient; SE, Standard Error; Bootstrap 95% CI, Bootstrap 95% Confidence Interval; LLCI, Lower Limit of Confidence Interval; ULCI, Upper Limit of Confidence Interval; R^2^, Coefficient of Determination; SS, Social Support; PA, Physical Activity; PsyCap, Psychological Capital; AB, Academic Burnout.

**Table 6:** Analysis of intermediation effects.

Effect	Path	*β*	Percentage	Bootstrap 95% CI	
				LLCI	ULCI
Total effect	PA → AB	−0.228	100%	−0.262	−0.195
Direct effect	PA → AB	−0.119	52.2%	−0.149	−0.090
Total indirect effects		−0.109	47.8%	−0.134	−0.086
Indirect effect	PA → SS → AB	−0.033	14.5%	−0.053	−0.017
	PA → PsyCap → AB	−0.023	10.1%	−0.035	−0.013
	PA → SS → PsyCap → AB	−0.053	23.2%	−0.072	−0.036

β, Beta Coefficient; Bootstrap 95% CI, Bootstrap 95% Confidence Interval; LLCI, Lower Limit of Confidence Interval; ULCI, Upper Limit of Confidence Interval; SS, Social Support; PA, Physical Activity; PsyCap, Psychological Capital; AB – Academic Burnout.

The regression analysis results revealed that PA was significantly positively correlated with SS (*β* = 0.129, *p* < 0.001) and PsyCap (*β* = 0.053, *p* < 0.001) and significantly negatively correlated with AB (*β* = −0.120, *p* < 0.001). Additionally, SS was significantly positively correlated with PsyCap (*β* = 0.952, *p* < 0.001) and significantly negatively correlated with AB (*β* = −0.260, *p* < 0.001). Furthermore, PsyCap was significantly negatively correlated with AB (*β* = −0.431, *p* < 0.001).

As shown in Table 6, the direct and total effects of PA on AB, as well as the indirect effects through SS and PsyCap, were examined. The results indicated that both SS and PsyCap independently mediated the relationship between PA and AB. When SS acted as a mediator, the indirect effect was −0.033, accounting for 14.5% of the total effect; when PsyCap acted as a mediator, the indirect effect was −0.023, accounting for 10.1% of the total effect, thus supporting H2 and H3. In addition, the study confirmed a chain mediation effect of SS and PsyCap between PA and AB, with an effect size of −0.053, accounting for 23.2% of the total effect, thereby supporting H4.

### 3.6 Moderating effects analysis

**Figure 2:**
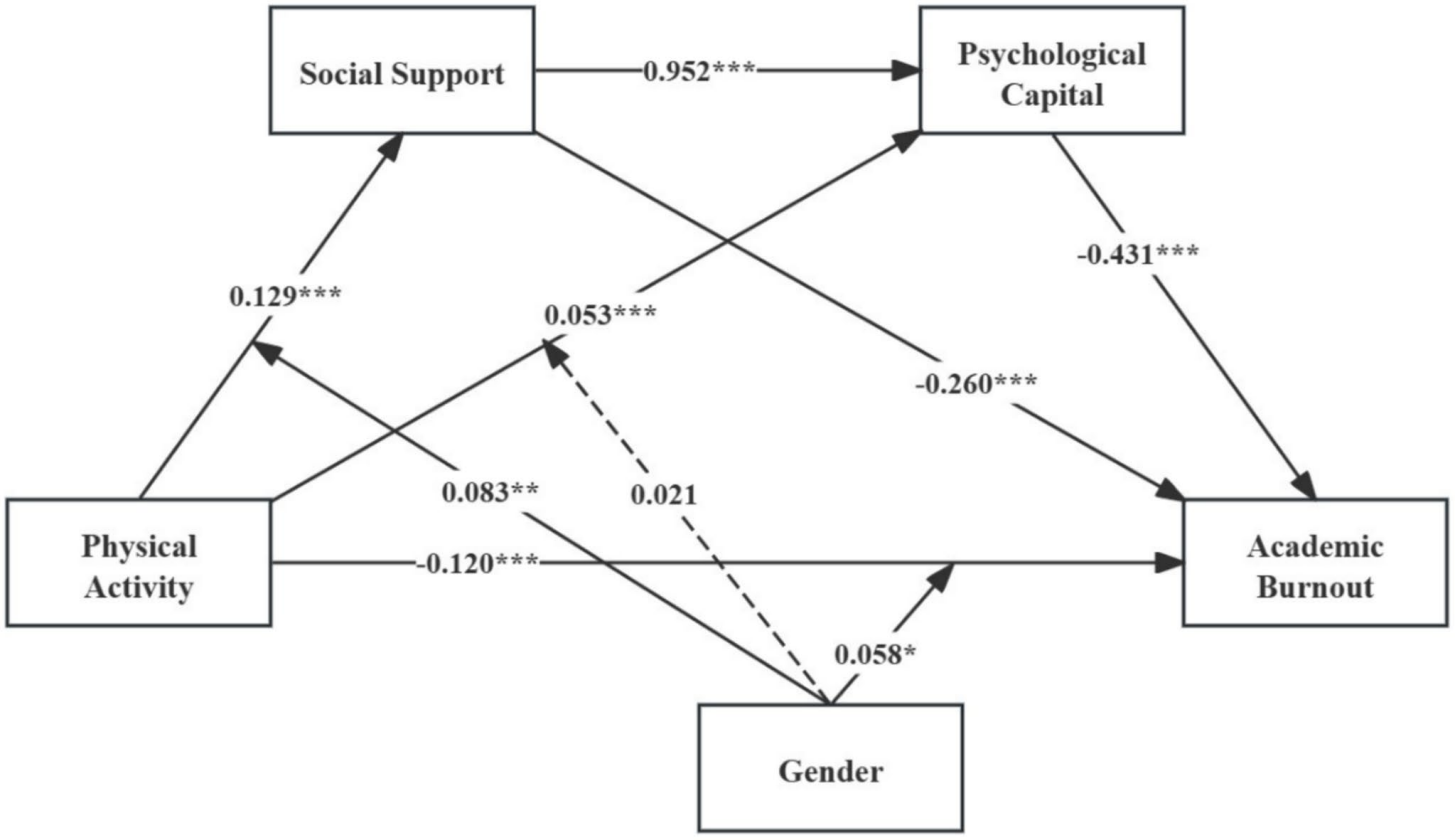
The moderated chain mediation model. *p < 0.05; **p < 0.01; ***p < 0.001. The solid lines indicate significant paths; the dashed lines indicate non-significant paths.

The moderated mediation analysis of gender was conducted using Model 85 of the SPSS PROCESS Macro version 4.2. The results are presented in Table 7 and Figure 2.

**Table 7:** Analysis of the mediating effect of regulation.

Outcome variable	Predictor variable	*β*	*p*	SE	Bootstrap 95% CI	
					LLCI	ULCI
SS	PA × Gender	0.083	0.001	0.026	0.032	0.134
PsyCap	PA × Gender	−0.021	0.317	0.021	−0.062	0.020
AB	PA × Gender	0.058	0.040	0.028	0.003	0.113

β, Beta Coefficient; SE, Standard Error; Bootstrap 95% CI, Bootstrap 95% Confidence Interval; LLCI, Lower Limit of Confidence Interval; ULCI, Upper Limit of Confidence Interval; SS, Social Support; PA, Physical Activity; PsyCap, Psychological Capital; AB, Academic Burnout.

**Figure 3:**
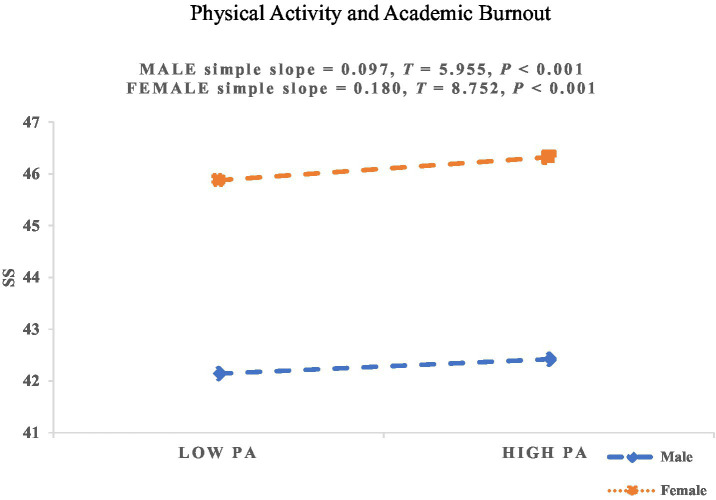
The moderating effect of gender on the relationship between PA and SS. SS, Social Support; PA, Physical Activity.

**Figure 4:**
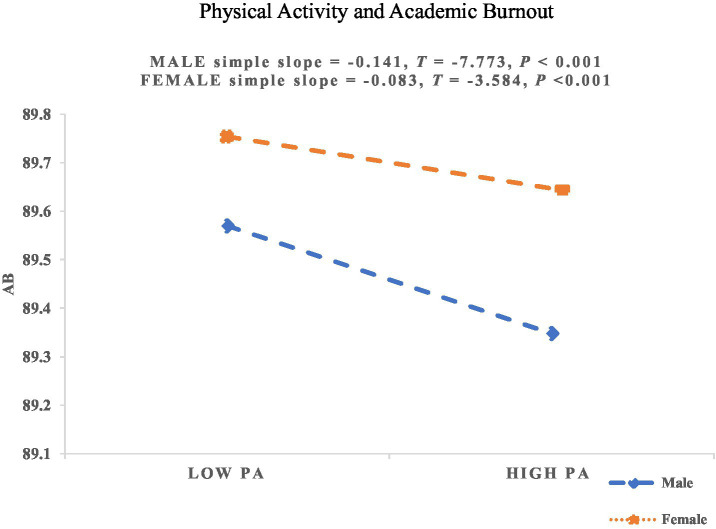
The moderating effect of gender on the relationship between PA and AB. PA, Physical Activity; AB, Academic Burnout.

As shown in Table 7, gender significantly moderated the relationships between PA and SS, as well as between PA and AB. However, gender did not significantly moderate the association between PA and PsyCap. Therefore, H5a and H5c were supported, while H5b was not supported. To further illustrate these moderating effects, simple slope plots were generated for the moderating role of gender on the relationship between PA and SS, as well as between PA and AB, as shown in Figures 3, 4.

Figure 3 illustrates a significant gender-based moderation in the PA–SS relationship, thereby supporting H5a. With increasing levels of PA, female college students (*β_simple_* = 0.180; *p* < 0.001) experienced a faster increase in perceived SS compared to male college students (*β_simple_* = 0.097; *p* < 0.001). These results indicate that as PA rises, female students are more prone to receiving higher levels of social support, demonstrating that PA contributes more significantly to perceived SS among females than males.

As shown in Figure 4, gender significantly moderated the relationship between PA and AB, supporting H5c. With increasing levels of PA, male college students (*β_simple_* = −0.141; *p* < 0.001) experienced a faster reduction in AB compared to female college students (*β_simple_* = −0.083; *p* < 0.001). This suggests that at higher levels of PA, males are more effective in reducing AB, indicating a stronger ability among male students to alleviate AB through PA compared to female students.

## 4 Discussion

Integrating stress-coping theory and gender role theory, the present study assessed the correlation between PA and AB among college students, highlighting the potential chain mediating functions of SS and PsyCap and the moderating influence of gender within this framework. Using PROCESS models in SPSS for data analysis, most of the proposed hypotheses were supported. The following sections discuss the research findings in detail.

The study identified a significant negative correlation between PA and AB. Consistent with this, Fu et al. (2023) observed that students who regularly engaged in PA tended to report lower levels of AB, suggesting that PA may, in certain contexts, help students manage academic stress. On the one hand, regular PA has been linked to increased cerebral blood flow and alters neurotransmitter levels (e.g., norepinephrine, endorphins, and serotonin), thereby augmenting stress regulation capacity (Chiappelli et al., 2023; Kleinloog et al., 2023). On the other hand, setting and achieving exercise goals may provide positive feedback in personal competence and confidence, boosts self-esteem, and reduces anxiety and depression, thus psychologically strengthening students’ ability to overcome academic challenges (Herbert et al., 2020; Tian and Yang, 2024). Therefore, participation in PA appears to be associated with reduced tension and lower both stress levels and AB among college students (Cairney et al., 2014).

This study further indicated that SS meditates the association between PA and AB, accounting for 14.5% of the total effect. This result is consistent with Gao et al. (2025), who found that PA enhances adolescents’ perceived SS, thereby reducing AB. According to social support theory (Cohen and Wills, 1985), individuals acquire various forms of support through interactions with others, groups, and institutions, which enhance both health outcomes and behavioral responses. Physical activity may serves as a vehicle for social interaction, creating opportunities for students to expand their support networks (Shu et al., 2024). Team sports and group activities, such as basketball, volleyball, and soccer, provide students with spaces for communication, fostering both emotional and instrumental support (Maher et al., 2024). Stronger SS is often associated with lower levels of AB by dampening stress responses, enhancing belongingness and security, minimizing psychological distress, and preventing burnout development (Ye et al., 2021). Furthermore, positive evaluations from external sources support the development of an enhanced self-concept, which in turn is linked to lower AB (Halbesleben and Ronald Buckley, 2006).

The study also found that PsyCap mediates the relationship between PA and AB, accounting for 10.1% of the total effect. Psychological capital theory (Luthans et al., 2006) posits that PsyCap is a key resource for coping with stress and challenges. Physical activity, as a positive lifestyle practice, can enhance PsyCap through multiple pathways (Lee et al., 2022). For example, PA may foster confidence and self-belief and has been associated with neurochemical processes, such as endorphin release, that may contribute to optimism and positive emotions (Anderson and Feldman, 2020). Moreover, PA enhances individuals’ resilience and nurtures hope, which empowers them to remain positive and employ more constructive methods to handle difficulties and stressful circumstances (Yao et al., 2022). Therefore, PA can enhance the PsyCap of college students across multiple dimensions. In the academic context, the development of PsyCap is particularly important, as it not only reflects students’ self-efficacy, hope, optimism, and resilience formed during the learning process, but also directly influences how they cope with academic stress and challenges (Carmona-Halty et al., 2019; Luthans and Youssef-Morgan, 2017). Previous studies have shown that high levels of PsyCap can significantly buffer the negative effects of learning stress on AB and play a central mediating role between academic adjustment and academic satisfaction (Elom et al., 2023; Virga et al., 2020). Increased PsyCap allows students to sustain positive mental states, strengthen emotional regulation, and better manage academic stress, thereby reducing emotional exhaustion, academic frustration, and feelings of low accomplishment, which are core symptoms of AB (Guo et al., 2023; Maslach et al., 1997).

Finally, this study identified a significant chain mediating effect of SS and PsyCap linking PA to AB, which constituted the largest component of the total effect, reaching 23.2%. First, this finding confirms the positive association between SS and PsyCap, consistent with Wang et al. (2023), who found that students receiving higher levels of emotional support and positive feedback are more likely to develop stronger PsyCap, including enhanced resilience, optimism, and hope (Luthans et al., 2006). Second, the bootstrap analysis confirmed the chain mediation effect, offering new insights into how PA alleviates AB. Regular participation in PA has been associated with opportunities to build stronger social networks and increase their access to emotional and informational support (Shu et al., 2024). Support from family and friends provides rich resources for the development of PsyCap (Wang et al., 2023). Ultimately, a higher degree of PsyCap enables students to better handle academic challenges, resulting in decreased anxiety, reduced emotional strain, and fewer signs of burnout (Zhao H. et al., 2025). In sum, SS and PsyCap play central roles in the chain mediation pathway between PA and AB, although these pathways should be interpreted as tentative and require longitudinal or experimental research for further verification.

The results indicated that gender significantly moderated the relationships between PA and SS, as well as between PA and AB, supporting H5a and H5c. This study found that females appeared more likely to report increases in SS through PA, whereas males showed a stronger association between PA and lower levels of AB. The current findings corroborate the conclusions reached in the works of Shu et al. (2024) and Chen et al. (2022). Females often participate in group-based or socially oriented physical activities, which may promote interpersonal interactions and emotional exchanges (Day and Livingstone, 2003). Consequently, they may be more likely to perceive PA as a means to acquire SS, which could explain their stronger association between PA and SS (Eschenbeck et al., 2007). In contrast, males frequently emphasize personal achievement and self-improvement during PA, which helps them strengthen their sense of control over academic tasks and reduce feelings of burnout (Matud, 2004; Schöndube et al., 2017). Furthermore, this difference may stem from the influence of gender role socialization and cultural expectations. Traditional social norms emphasize women’s roles in relationship maintenance and social bonding, making them more likely to associate PA with the acquisition of SS. In contrast, men are often expected to demonstrate independence and achievement orientation, which leads them to rely more on PA as a way to strengthen coping ability and a sense of control when facing academic stress. However, existing research is not entirely consistent. Some studies suggest that women benefit more from SS than men, which is linked to their interaction patterns within social networks (Bedrov and Gable, 2023; Perrewé and Carlson, 2002). Other scholars, by contrast, have found that men more frequently adopt behavioral coping strategies, such as PA, when dealing with stress, and that such strategies may be more effective in alleviating negative emotions and academic stress (Wijndaele et al., 2007). Therefore, the moderating role of gender in these two pathways may originate from fundamental differences in coping strategies between men and women. These contradictory findings suggest that gender’s moderating role in the relationships between PA and SS and between PA and AB is shaped not only by sociocultural expectations but also by individual differences and contextual factors, thereby pointing to a more complex mechanism that warrants further investigation.

Gender was not identified as a significant moderator in the relationship between PA and PsyCap in this study; therefore, H5b was not supported. This may be because PA, as a relatively neutral form of positive behavior, enhances PsyCap primarily through individual factors such as participation level, engagement, and the quality of the experience, rather than gender itself. The self-efficacy, positive emotions, and sense of achievement generated by PA may exert similar effects across genders, thereby attenuating the moderating effect of gender on this pathway. Taken together, these findings point to plausible pathways that should be interpreted cautiously and verified in future longitudinal or experimental research.

## 5 Implications

Grounded in stress-coping theory and gender role theory, aimed to identify factors mitigating AB among college students and to clarify the associations linking these factors. The findings offer important theoretical and practical implications, providing new insights into promoting college students’ psychological well-being and academic development.

### 5.1 Theoretical implications

First, by introducing PsyCap and SS as chain mediators, this study demonstrated that PA impacts AB both directly and indirectly by increasing perceived SS, which subsequently promotes PsyCap. This expanded perspective deepens the theoretical framework of AB and offers a more systematic pathway analysis for understanding how PA impacts AB. Second, this study successfully integrated stress-coping theory and gender role theory. Stress-coping theory emphasizes the influence of individuals’ coping strategies on psychological well-being when facing academic stress, while gender role theory underscores how culturally prescribed gender norms guide individuals’ behavioral responses and psychological outcomes. The integration of these two theoretical perspectives provides a comprehensive understanding of gender differences in how PA and SS contribute to the development of PsyCap, thereby enriching the theoretical framework of AB. Finally, this study focused on assessing the moderating influence of gender on the interrelations among PA, SS, and AB. The study found that female college students are more effective in gaining SS through PA, whereas male college students experience a stronger effect of PA in alleviating AB. This evidence advances theoretical frameworks concerning the influence of gender-related factors on psychological well-being. These findings not only reveal the role of gender in the relationships among PA, SS and AB, but also offer new directions for further research on gender differences in mental health.

### 5.2 Practical implications

First, the conclusions drawn from this study provide an effective pathway for college students to alleviate AB and promote self-improvement. Based on these results, students can clearly recognize the interconnections among PA, SS, and PsyCap. By actively expanding their social networks during PA and consciously cultivating positive psychological qualities, students can establish a virtuous cycle of “PA—acquiring SS—enhancing PsyCap—alleviating AB,” thereby achieving a balanced development of both mental health and academic performance.

Second, for educators, the findings offer new perspectives for teaching practice and student guidance. Teachers may incorporate PA into daily instruction, such as integrating team-building exercises into group projects, which can foster trust and relieve academic stress through collaborative physical tasks. For example, female students may benefit more from group-oriented and interactive activities (such as group fitness, dance, or team sports), which can enhance their experiences of SS. In contrast, for male students, more challenging and achievement-oriented physical activities (such as competitive sports or extreme sports) can be designed to help transform the perseverance fostered through exercise into academic motivation. In terms of mental health education, educators can conduct workshops and lectures to disseminate knowledge on using PA to mitigate AB, helping students develop exercise habits and adopt healthy lifestyles.

Finally, at a more macro level, university administrators and policymakers should also recognize the importance of PA in mitigating AB. This study suggests that universities can strengthen physical education within talent cultivation systems by ensuring sufficient credits for compulsory PE courses, offering interdisciplinary electives such as sport psychology and health management, and creating a strong athletic culture through events like campus marathons and sports festivals. These initiatives provide students with more opportunities for social interaction and the development of PsyCap. Meanwhile, government bodies can support nationwide PA promotion programs through policy and funding, such as increasing investment in university sports infrastructure, sponsoring diverse student sports clubs, and launching public campaigns that integrate PA with mental health education. Embedding PA and mental health promotion into national and institutional strategies can help enhance students’ psychological resources on a larger scale, reduce AB, and foster holistic development.

To make these recommendations more actionable, three concrete measures can be emphasized. First, universities should track participation rates in structured PA programs (e.g., sports clubs, physical exercise classes, campus events) as a direct indicator of student engagement. Second, institutions should regularly monitor students’ AB scores using validated burnout scales to assess whether PA initiatives are reducing exhaustion, cynicism, and low efficacy. Third, universities can evaluate PsyCap scores before and after targeted mentoring, feedback-based learning, or challenge-oriented PA programs to determine whether students’ self-efficacy, resilience, optimism, and hope are being strengthened. These measurable indicators allow institutions and policymakers to monitor progress and adjust interventions effectively.

## 6 Limitations and future research

Although this research offers theoretical contributions and empirical findings the correlation between PA and AB among college students, certain limitations remain.

First, the study’s reliance on cross-sectional data collection, conducted at one moment in time, constrains the ability to determine cause-and-effect relationships among PA, SS, PsyCap, and AB. Although the study examined correlations among these variables, it was unable to capture the dynamic changes that may occur over time, leaving open the possibility of unmeasured variables or reverse causality influencing the results. Future research may implement longitudinal research frameworks to better understand the evolving causal dynamics among these constructs, particularly the sustained impact of PA on AB. For instance, longitudinal designs could further explore the long-term effects of PA on AB and examine whether the cumulative effects of PsyCap over time become more pronounced.

Second, the use of self-administered survey instruments may have introduced response biases, such as social desirability tendencies or recall inaccuracies, potentially affecting data validity. Due to the sensitive nature of topics concerning mental health and personal habits, participants may have responded in a socially favorable manner, potentially affecting the objectivity and credibility of the data. To reduce such risks, the survey was conducted anonymously and participants were assured that their responses would be kept strictly confidential and used only for research purposes. These measures helped minimize social desirability effects to some extent. To further improve reliability and validity, future research could adopt multi-source data collection approaches, including physiological monitoring, direct behavioral observations, and informant reports from teachers or peers, to improve the reliability and validity of findings.

Third, despite identifying gender’s moderating role in the relationships between PA, SS, and AB, the study did not systematically uncover the underlying processes responsible for these gender disparities. The analysis treated gender as a single categorical variable and overlooked additional factors that could contribute to gender differences, such as gender role socialization, cultural background, or individual gender identity. Future research could analyze in greater detail how gender roles, through socialization processes, influence the functions of PA. For example, in cultures that emphasize group support, women may be more likely to use PA as a means of acquiring SS, whereas in cultures that emphasize individual achievement, men may be more inclined to leverage PA to enhance PsyCap as a way of alleviating AB. Cross-cultural studies may also help to reveal similarities and differences in the relationships among PA, SS, and PsyCap across diverse social environments, thereby enriching the understanding of gender-based mechanisms.

Fourth, the study did not collect detailed demographic information, such as participants’ academic major, socioeconomic status, or geographic background. Previous research has shown that factors such as major (e.g., STEM fields) and academic performance are important determinants of AB (Liu et al., 2023; Turhan et al., 2023). Socioeconomic background and regional differences may also influence how students utilize resources and perceive stress. Therefore, the generalizability of the findings remains limited. Future research could build on this study by investigating how demographic characteristics shape the relationships examined in the model.

Fifth, although gender, age, and grade were controlled in the regression analyses, other potential confounding variables were not included, such as students’ workload, prior mental health history, or family socioeconomic background. This limitation may affect the robustness of the conclusions. Future studies should incorporate larger and more diverse samples, along with richer demographic indicators and additional control variables, to more comprehensively validate the generalizability and robustness of the relationships among PA, SS, PsyCap, and AB.

Finally, the study did not record the specific types of PA in which participants engaged, which limits the ability to verify whether previously reported gender-specific patterns (e.g., men preferring soccer or basketball, women preferring yoga or dance) were also present in this sample. More broadly, inconsistent findings in the literature may be partly explained by moderators such as the intensity and type of PA, whether social support is assessed as perceived or actually received, and cultural norms that shape both activity participation and coping strategies. Future research could employ more fine-grained measurement tools, longitudinal frameworks, or mixed-methods designs to examine not only gender- and culture-specific differences in PA preferences but also how these differences influence the SS and PsyCap pathways in relation to AB.

## 7 Conclusion

Academic burnout has become increasingly common in higher education, and identifying ways to address burnout caused by academic stress has drawn significant scholarly attention. This study explored how PA, through the mediating roles of SS and PsyCap, is associated with lower levels of AB among college students. Utilizing self-reported information from 858 participants, the study found a significant negative association between PA and AB, with SS and PsyCap serving as both independent and serial mediators. The findings suggest that PA may be linked to higher perceived SS and stronger PsyCap, which in turn could relate to lower AB. In addition, gender differences were identified: PA showed a stronger association with SS among female students, while male students reported a stronger association between PA and reduced AB. The current findings expand theoretical knowledge of the links between PA and AB, while offering practical implications for educators and academic organizations. The reliance on self-reported tools and cross-sectional design constitutes a notable methodological limitation that should be acknowledged. Future studies are encouraged to expand on these results and carry out more detailed and thorough investigations.

## Data Availability

The datasets presented in this study can be found in online repositories. The names of the repository/repositories and accession number(s) can be found in the article/supplementary material.

## Footnotes

^1^ https://www.wjx.cn/
